# Reframing infection control approaches in low-resource health care settings: A nod to the emic perspective

**DOI:** 10.7189/jogh.10.020340

**Published:** 2020-12

**Authors:** Samantha J Sadler, Anthony T Fuller

**Affiliations:** 1 Duke Global Neurosurgery & Neurology, Durham, North Carolina, USA; 2 Harvard Medical School, Boston, Massachusetts, USA; 3 Duke University, Durham, North Carolina, USA; 4 Duke University, Department of Neurosurgery, Durham, North Carolina, USA

Healthcare-associated infections (HCAIs) comprise a significant public health concern that, like many of the world’s communicable diseases, preys on the most vulnerable. Compared to high-income countries (HICs), low- and middle-income countries (LMICs) are vulnerable to higher HCAI rates, which are associated with worse patient outcomes and excess costs [1].

The recent outbreak of Severe Acute Respiratory Syndrome-Coronavirus-2 (SARS-CoV-2) threatens to exacerbate these inequities, with LMICs facing contextual challenges to controlling the virus that may limit the effectiveness of recommended approaches [2] For example, although early studies have suggested infection control efforts such as early quarantine efforts may be effective in slowing the spread of this global pandemic [3], the ability to comply with these strict rules often hinges upon financial and/or structural privileges of individuals and/or institutions. This is especially true for health care institutions in LMICs that may have limited access to the recommended equipment for preventing or slowing the spread of SARS-CoV-2 within their facilities. As heightened HCAI rates constitute a major, multifaceted burden in LMICs, SARS-CoV-2 highlights how improving infection control practices in LMIC health care settings is essential to reducing infections, alleviating the financial burden on vulnerable, economically developing LMIC health care systems, and ultimately achieving more equitable outcomes [1].

## REFRAMING INFECTION CONTROL APPROACHES IN LOW- AND MIDDLE-INCOME SETTINGS

As studies begin producing recommendations for controlling SARS-CoV-2 in low-resource settings, it should be recognized that effective implementation of evidence-based infection control practices remains a challenge. In addition to inadequate financial and material resources, a combination of overcrowding, understaffing and weak governance structures can render the execution of proper infection control techniques infeasible [1,4].

Tailoring infection control or other quality improvement interventions may enhance project impact [4,5]. Gardam and colleagues have explored how local, context-specific social and cultural norms can shape the efficacy of an infection control intervention; moreover, choosing an infection control *strategy* that emphasizes local norms may foster better outcomes [6] A review of efforts to implement WHO clinical guidelines for hospital care in LMICs likewise correlated efforts to adapt the clinical guidelines to the local setting with improved implementation results [5]. Therefore, in addition to respecting broader, systemic limitations of an LMIC setting, both the intervention design and implementation methods may benefit from contextual adaptation at the *local* level.

**Figure 1:**
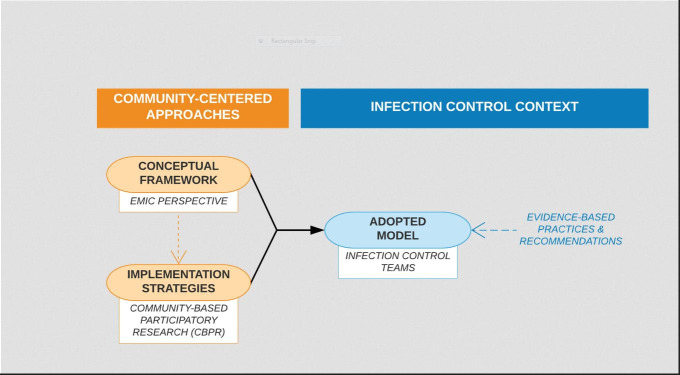
Reframing infection control approaches.

Therefore, in determining how to most effectively navigate these challenges to producing effective infection control strategies in LMIC settings, researchers should (first) adopt a locally-centered **conceptual framework** to ensure the foundation of the approach is culturally- and contextually-informed, (second) identify evidence-based **implementation strategies** that reflect the goals of the locally-centered conceptual framework, and then (third) identify an **implementation model** to effectively apply those evidence-based, locally-centered strategies in the context of infection control practices (**Figure 1**).

### I. Framework: the emic perspective

Pike (1967) coined two different anthropological perspectives in seeking to understand context with respect to culture: the *emic* perspective, which emphasizes studying behavior from *within* the given culture itself, vs the *etic* perspective, which involves studying behavior from an external, alien viewpoint outside of the given culture (Pike, as cited in Berry (1989); see for a more detailed explanation) [7] In other words, the emic perspective “clarifies intrinsic cultural distinctions,” whereas the etic perspective “seeks objectivity as an outside observer across cultures” [8].

This dichotomy can be applied to intervention approaches: creating an intervention that, at its core, prioritizes local application involves taking an *emic* approach, one which is wrought from within the perspective of the local culture, rather than an *etic* perspective, which is one generated from an outside perspective and applied to an alternative culture. In this way, emic, locally driven approaches have the benefit of greater contextual and cultural compatibility, which - as described here - may be important to effectively implementing interventions across different contexts.

### II. Strategies: community-based participatory research

By definition, local stakeholder involvement and authority is tied to facilitating an emic perspective. To properly inform local adaptation, studies have called for active local stakeholder engagement in the design and implementation of infection control projects [9] Community-based participatory research (CBPR) is a good example of systematizing this; key to CBPR is an equal sharing of responsibility between researchers and community partners, wherein both maintain the right to provide input on the project with the promise of thoughtful consideration by the other [10]. In this way, CBPR exemplifies the potential efficacy of a local team where the responsibility and authority ultimately rests with the local stakeholders, particularly in terms of project development and implementation [11]. Therefore, researchers should draw lessons from CBPR studies and team models to systematically develop and implement locally driven infection control efforts.

**Figure:**
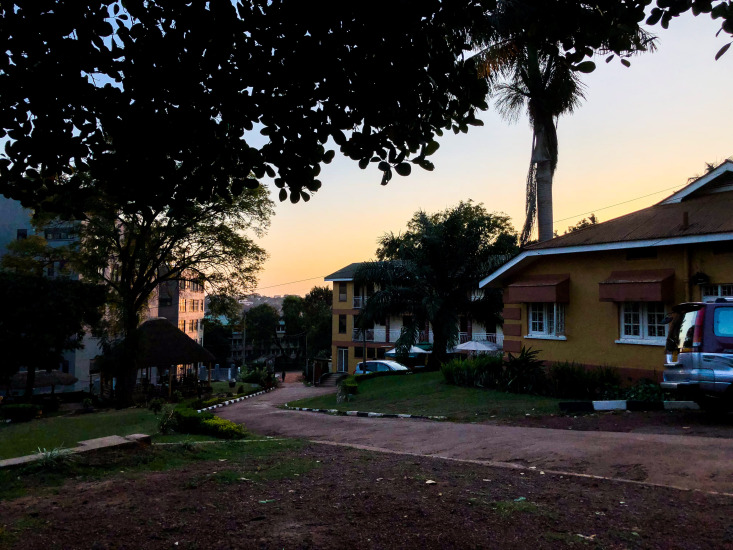
Photo: Guest house at Mulago National Referral Hospital in Kampala, Uganda (from Samantha Sadler’s collection, used with permission).

### III. Adapted Model: the infection control team

Local infection control and prevention programs run by a dedicated team have been identified as important to infection control in low-resource settings [4]. However, the utility of these local teams may be limited by the fixed, “one-size-fits-all” infection control recommendations for LMICs themselves. For example, Storr et al. (2017) noted that a general weakness to the WHO’s infection control guidelines (2016) was that the guidelines could not be considered comprehensive due to the infinite number of possible situations diverse health care settings may face [12]. By adhering to models proven effective in other contexts and thereby imposing these external, etic regulations diminishes the uniquely emic potential of a local infection control team. Moreover, this highlights a trend in infection control research of broadly grouping low-resource settings in a way that eclipses inter-setting diversity to the detriment of local communities.

If properly empowered, the **local infection control team (one consisting of and directed by local stakeholders)** could address these contextual gaps in infection control intervention design and implementation; through constructing their own adapted infection control initiatives, outcomes might improve by nature of greater local relevance. Limited studies have explored systematically increasing local input in infection control efforts. For example, a study conducted in a Brazilian hospital found that by implementing a twice-monthly meeting for local health care workers to discuss their perceptions of and suggestions to improve hand hygiene, compliance to proper hand hygiene increased, and associated infection rates decreased [13] An example of intentionally leveraging the emic perspective in infection control efforts lies in a program implemented by Duke Global Neurosurgery & Neurology (DGNN) in Kampala, Uganda; the Neurosurgery Infection Control Team (NICT) was developed among Mulago National Referral Hospital (MNRH) neurosurgery ward staff as a case study examining the feasibility and acceptability of a local infection control team in terms of performance effectiveness and attitudinal and behavioral outcomes. Preliminary results suggest a high degree of self-efficacy and empowerment among NICT members, as well as improved infection rates. A more in-depth analysis is forthcoming [14] A study conducted in Columbia used a similar method of guided project development; two separate infection control teams each consisting of local hospital personnel were encouraged to design a post-cesarean infection reduction approach, and each successfully improved clinical outcomes through their different proposed methods [15] Through applying and systematizing such community-based participation, a designated local infection team consisting of local staff familiar with ward norms could systematically facilitate the production and implementation of such locally-informed approaches.

Moreover, the CBPR implementation model explored above may help local infection control teams navigate financial and infrastructural barriers that may hinder their effectiveness. However, such a partnership must be pursued with great caution; the CBPR principle of local stakeholder empowerment must be prioritized to ensure any outside provision of support does not perpetuate historical power differentials and cause undue dependability on external providers.

## CONCLUSION

An intervention is only as effective as its implementation. As the latter remains a challenge in low-resource settings due to financial, infrastructural and other contextual challenges, explicit consideration must be given to adapting SARS-CoV-2 infection control practices to local contexts. Through implementation strategies prioritizing local stakeholder empowerment (eg, CBPR-derived), the local infection control team model could be feasibly adapted to facilitate locally informed (and thereby emic) infection control approaches.

Few studies have assessed ways in which such *local* infection control teams might provide the flexibility needed to ensure infection control practices are adopted while ensuring efficacy of the infection control practices in place. Future studies should further investigate the trade-offs between prioritizing local specificity over existing evidence-based practices and outcomes in terms of local stakeholder perspective, infection control rates, and relative sustainability of implementation and change.

SARS-CoV-2 threatens to exacerbate the already disparate burden of HCAIs in low-resource settings, highlighting the urgent need to improve infection control efforts in these facilities. As researchers consider ways to best combat this spread, such approaches must prioritize the emic perspective to maximize local empowerment and representation during this time of global need.
